# Breastfeeding support at an Australian Breastfeeding Association drop-in service: a descriptive survey

**DOI:** 10.1186/s13006-020-00345-1

**Published:** 2020-11-30

**Authors:** Elaine S. Burns, Louise Duursma, Zoi Triandafilidis

**Affiliations:** School of Nursing and Midwifery, Western Sydney University, Sydney, New South Wales Australia

**Keywords:** Breastfeeding, Lactation, Peer support, Peer counsellor, Thematic analysis, Reactive support, Drop-in

## Abstract

**Background:**

In Australia, during the early establishment phase of breastfeeding, women can access telephone peer support counselling provided by the Australian Breastfeeding Association (ABA) however options for face-to-face peer support are limited. The known factors which improve ongoing and exclusive breastfeeding include face-to-face support, peer and/or professional support, and trained personnel. This study aimed to examine women’s experiences of accessing one breastfeeding drop-in peer support service provided by trained peer support volunteer counsellors from the ABA.

**Methods:**

Women who accessed the service were invited, in 2014, to participate in an anonymous online survey which collected both quantitative and qualitative data. Participants were asked about their experiences of breastfeeding support, as well as their experiences of the drop-in service. In total, 53 women completed the online survey, and subsequent analysis generated descriptive statistics and qualitative themes.

**Results:**

Responses to the survey revealed that women attended the drop-in service with infants ranging in age from less than 1 week through to 12 months of age. Most women reported attending with infants aged 0–8 weeks of age (72%). The predominant presenting problems identified were sore/damaged nipples, difficulties with infant latching to the breast, or concerns about using nipple shields. Analysis of the open text qualitative responses revealed one overarching theme ‘Support to continue breastfeeding’ and four subthemes: ‘feeling listened to and not judged’; ‘emotional support and confidence building’; ‘the importance of face-to-face, practical support’; and ‘the need for ongoing, free access’.

**Discussion:**

In this study many women were seeking support for ongoing breastfeeding difficulties. Health professionals who had limited breastfeeding knowledge and skills were identified as most unhelpful in providing support with ongoing breastfeeding difficulties. Women valued having access to trained peer counsellors, who had the capacity to provide non-judgemental, face-to-face support; who could sit through a feed; in a space that was ‘safe’; and who could enhance a woman’s confidence with breastfeeding over the course of her full breastfeeding journey.

**Conclusions:**

Reactive peer support, provided in response to need, at an Australian Breastfeeding Association drop-in service, was described by participants as pivotal to enabling their ongoing breastfeeding.

## Background

It is well established that breastfeeding is important for the short and long term health and wellbeing of mothers and babies [1], however, many Australian women do not meet global benchmarks [2], nor their own personal breastfeeding goals [3]. Women report, both in Australia [4] and internationally [5–7], that early breastfeeding support from health professionals is insufficient, inadequate, at times inappropriate and ceases after the first couple of weeks [8]. The latest Cochrane review (2017) [9], on support for breastfeeding mothers, states that the factors which improve ongoing and exclusive breastfeeding include face to face support, peer and/or professional support, and trained personnel. A systematic review conducted by Shakya and colleagues [10] showed that, in high income countries, community-based peer support was effective in increasing exclusive breastfeeding rates at 3 months, when compared to standard care.

The Australian Breastfeeding Association (ABA) is a registered training organisation and the largest information and breastfeeding support service for the community, new mothers, and health professionals, in Australia [11]. ABA breastfeeding counsellors are mothers who have breastfed and completed 900 h of breastfeeding education and counsellor training [12]. ABA peer volunteers provide reassurance and up-to-date information to women about breastfeeding, as well as practical tips and suggestions [13]. ABA peer counselling is based on a Rogerian model, which is a relationship-based model of care, and includes communicating empathy, building rapport and having a non-judgemental positive regard for the woman [14]. ABA predominantly provides support through their 24 h telephone helpline, and online or in community group meetings [15, 16]. Face to face peer support with a trained ABA counsellor is provided on an ad hoc basis.

The aim of this study was to examine women’s experiences of accessing a peer support drop-in service provided by trained ABA volunteer peer support counsellors. This ABA drop-in service operated for 2 years between 2012 and 2013, in Sydney, Australia. Despite the growth in women accessing the service it was closed due to State government funding uncertainty. Women were referred to the drop-in service by health professionals, by ABA peer counsellors, or they found out about the service by word of mouth. The online survey was collected 8 months after the service had closed in August–September 2014.

### Setting

The ABA drop-in breastfeeding peer support service (hereafter referred to as the drop-in service) was located in North-Western Sydney in the office of the New South Wales (NSW) branch of the ABA. It was the only available ABA drop-in service in NSW, and there has not been another since it ceased operating in 2014. The office was the centre of many ABA activities including administration, volunteer training and breastfeeding peer support group meetings. It was staffed by ABA counsellors, in a voluntary capacity, and two paid administrative staff members, who were also ABA counsellors. The drop-in service aimed to provide a relaxed, home-like environment for women who required face-to-face support with breastfeeding.

The drop-in service operated 1 day per week, and no appointment was necessary. Women could attend as often as they needed, and consultations were not timed. Approximately 3–10 women accessed the service each week. The service was free for ABA members (yearly subscription cost $55). Non-ABA members were provided with one consultation and invited to join the ABA for ongoing support. There were no additional services provided other than breastfeeding support. For example: babies were not weighed or measured during their visit as parents were encouraged to maintain engagement with their local early child health services.

### Study design and methods

This study was a simple descriptive survey design consisting of 16 questions. The questions were developed by EB and LD to meet the study aims and sought to capture both quantitative and qualitative data on experiences using the drop-in service. The survey was pilot tested, by colleagues, for question clarity. Survey questions were mostly closed ended questions with multiple response options focusing on: reasons for seeking support; access to support prior to attending the drop-in service; satisfaction with the service and satisfaction with prior support options; and whether it mattered that the service was free. Due to a perceived high incidence of potential ‘tongue restriction’, noticed by the peer counsellors, there was also a question in the survey. Two open ended questions asked women to describe their experience of attending the Breastfeeding Drop-in Service and invited women to provide any additional feedback about the service.

Ethics approval for this study was obtained from Western Sydney University Human Research Ethics committee (H10852) and the Australian Breastfeeding Association (ABA Approval Number 2014–11). Women who attended the drop-in service over the two-year opening period and provided an email address (*n* = 136), were contacted 8 months after the service closed and invited to participate in an online survey. The participant information statement and survey link were emailed to participants, and the survey promoted online to ABA members in the local area, via local Facebook groups. All participants anonymously, and voluntarily, accessed the online survey. Completion of the survey implied consent and participants were able to provide as much or as little information as they deemed necessary.

### Data analysis

The quantitative survey data were analysed to generate descriptive statistics. Demographic data were collected on: how far women travelled to the service, their postcode, and how old their infant was when they attended the service. The open text qualitative data were thematically analysed using the Braun and Clarke [17] method. This is an inductive, iterative process of identifying patterns in the data. Data were first cleaned by ZT and any identifying information removed. Then a preliminary analysis was undertaken by ZT to find patterns and recurring themes in the data. EB and ZT then used Braun and Clarke’s six phase thematic analysis to develop themes from the data [17]. We progressed back and forth through the phases of thematic analysis firstly becoming familiar with the data, identifying initial codes, searching and reviewing themes and grouping the relevant data within each theme, and then eventually naming the themes [17]. These themes were developed and discussed by all authors until consensus was reached.

## Results

In total, 267 women accessed the drop-in service during the 2 years of operation and 136 women provided an email address. We contacted women by email and also promoted the survey on the local ABA Facebook groups. In total, 53 women accessed the online survey. Data were not included for two women who did not complete all questions, leaving 51 responses for analysis. Women indicated that their main reason for accessing the drop-in service was for assistance with breastfeeding, rather than to hire equipment or attend a group class or meeting. Most women lived within 30 mins of the service location (71%, *n* = 38) and others reported that they travelled up to an hour (25%, *n* = 13), and a small number travelled between 1 and 2 h (4% *n* = 2).

Responses to the survey revealed that women attended the drop-in service with infants ranging in age from less than 1 week through to 12 months of age. Most women attended with infants aged 0–8 weeks of age (72%, *n* = 37) and a significant number of presentations occurred at 0–4 weeks after birth (49%, *n* = 22). The most common presenting problems included sore/damaged nipples, difficulties with infant latching to the breast, or concerns about using nipple shields (see Table 1).

**Table 1:** Reasons for attending the Drop-in Service

If you attended for breastfeeding support what were the main reasons that you needed support? (select all applicable)	Responses *N* =
Tongue tie	31
Sore/damaged nipples	29
Baby unable to latch	27
Lip tie	19
Breast refusal	13
Low supply	12
Mastitis/block ducts	10
Concerns about baby’s weight	10
Over supply	8
Gastric reflux	8
Other (please specify)	8
Unsettled at the breast	6
Information	5
Sleep and settling issues	4
Blocked nipple pore (white spot)	2
Lactose intolerance	1
Return to work planning	1
Allergies	0
Help with weaning	0
Preparing for breastfeeding whilst pregnant	0
**Number of Participants who answered**	**51**

Prior to attending the service women sought breastfeeding support from a variety of other services including early child health nurse 61% (*n* = 31), lactation consultant at hospital 55% (*n* = 28), ABA helpline 41% (*n* = 21), family and friends 39% (*n* = 20) and the local General Practitioner (GP) 25% (*n* = 13) (see Table 2).

**Table 2:** Sources of support prior to attending the Breastfeeding Drop-in service

Sources of support prior to attending drop-in service (select all applicable)	Responses *N* =
Early Child Health Nurse	31
Lactation consultant at hospital	28
ABA Breastfeeding Helpline	21
Family and friends	20
GP/Local doctor	13
Local Australian Breastfeeding Association (ABA) Counsellor	10
Private lactation consultant	9
Paediatrician	7
Other (please specify)	7
Home midwifery	6
Osteopath	4
No one	3
Private midwife	3
Speech therapist	2
Chiropractor	2
Doula	1
Karitane	1
Tresillian	0
Naturopath	0
**Number of Participants who answered**	**51**

Participants were asked to rate a total of 18 potential sources of care, accessed prior to the drop-in service, based on a 4-point Likert scale ranging from not helpful to extremely helpful. Four groups were rated as most helpful prior to accessing the drop-in service: ABA breastfeeding helpline, lactation consultant at hospital, local ABA counsellor and family and friends, see Table 3. Some participants in this study rated the least helpful sources of breastfeeding support as Early Child Health Nurses, General Practitioner Doctors (GPs), Paediatricians and Lactation Consultants at hospital.

**Table 3:** Rating of Breastfeeding Support prior to attending the Drop-in service (condensed to Very/Extremely helpful and Not helpful)

Service provider	Very helpful or Extremely helpful	Not Helpful
ABA Breastfeeding Helpline	18	0
Lactation consultant at hospital	16	8
Local ABA Counsellor	13	0
Family and friends	12	5
Private lactation consultant	7	0
Early Child Health Nurse	6	13
Private midwife	4	0
Home midwifery	3	2
Other	3	0
Osteopath	2	1
Karitane	1	1
GP/Local doctor	0	11
Paediatrician	0	8
Doula	0	0
Speech therapist	0	1
Chiropractor	0	0
Tresillian	0	0
Naturopath	0	0
**Number of Participants who answered**	**51**	

Participants were asked whether the drop-in service was helpful, or not, based on the same 4-point Likert scale ranging from not helpful to extremely helpful. In total, 95% (*n* = 49) rated the service as ‘very helpful’ or ‘extremely helpful’. A large majority, 90% (*n* = 46) of women, reported that the ABA drop-in service helped them to achieve their breastfeeding goals. In particular, 70% (*n* = 36) of women, who responded to the survey, stated that their baby was referred to a doctor, as a result of the visit to the drop-in service, and that tongue-tie was eventually diagnosed. Whilst the peer counsellors could not diagnose tongue-tie, listening to women’s concerns enabled them to make appropriate referrals to health professionals for assessment. In 89% (*n* = 31) of cases the infants tongue-tie was released by a health professional. When asked about the important aspects of the drop-in service, 58% (*n* = 29) of women indicated that not having to pay for the drop-in service was either important or extremely important.

**Fig. 1:**
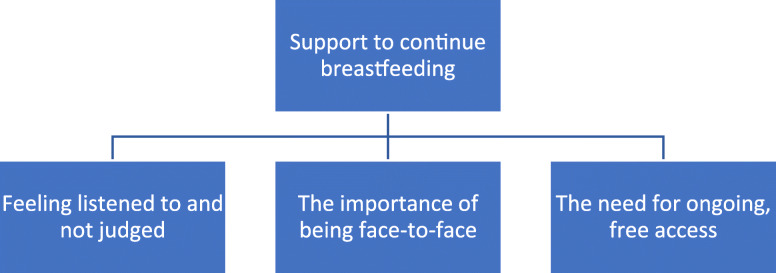
Overarching theme and subthemes

In total, 50 women responded to the invitation to provide open text responses to two questions. Data was uploaded, cleaned and coded using NVIVO 10. The overarching theme in relation to participants experiences of the drop-in service was ‘Support to continue breastfeeding’. Within this umbrella theme, three subthemes existed; ‘feeling listened to and not judged’; ‘the importance of face-to-face, practical and emotional support’; and ‘the need for ongoing, free access’. See Fig. 1.

### Support to continue breastfeeding: “Without that visit I would not have still been breastfeeding”

Women described the importance of the drop-in service availability and how the support enabled them to continue to breastfeed. The open text responses were full of positive responses about the ‘enabling’ nature of the drop-in service support for continuing breastfeeding, with one participant saying, “Without that visit I would not have still been breastfeeding” (Survey Participant (SP) 50).

Women disclosed that in some instances a problem that had been missed by various health practitioners had been picked up at the drop-in service, as illustrated by the following account:

They picked up the problem immediately and provided info and support and referred me to a lactation consultant … They were life savers and listened to me when I said there was something wrong. The [lactation consultant] I was later referred to discovered a 50% tongue tie and a 100% lip tie. Nobody else prior, out of all the people we saw, even noticed. (SP 42)

This type of response was common among respondents. Women stated that the drop-in service was unusual because “there is very little help out there for breastfeeding mothers” (SP 16). The practical nature of the help was appreciated by respondents of the survey, as the following quote reveals:

It is very difficult to get help from a GP or through a hospital if your baby is past the newborn stage. GP’s are not trained in lactation. And hospital-based lactation consultants are reluctant to see older babies. And private lactation consultants are expensive. Besides ABA, there is nowhere else for mums struggling to feed to go. (SP 19)

The weekly availability of the drop-in service helped women, who felt that without it they would have to wait for weeks to get help. Many women evidenced the value of the service by citing the length of time they had been able to continue to breastfeed, which ranged from six to 21 months, as these quotes highlight:

I was close to giving in [but] she is now 16 months and still breastfeeding. (SP 28)

I never would have kept going to 6 months without this free weekly support (SP 2)

Women described feeling a sense of “gratitude” to the peer counsellors, and ABA more generally, for their ongoing support, describing it as an “invaluable service”. This gratitude was shared by women who were unable to establish breastfeeding, but were supported to continue to feed their child breastmilk:

My daughter didn’t ever end up breastfeeding for more than a minute or two but thanks to the drop-in lounge and ABA support she had expressed breast milk for her first 4 months (SP39).

### Feeling listened to and not judged: “She was a good listener, not pushy and very respectful”

The non-judgemental nature of support was a repeated theme in the analysis of women’s responses. The absence of judgement was particularly important for women who were bottle feeding artificial formula to infants, as the following quote highlights:

I felt that it was a place that I could go to receive helpful advice and tips without being judged for bottle feeding my baby (SP 25).

The “non-judgemental” support at the drop-in service was described as caring and sensitive and women described being given the time to talk and feel listened to. For some women they felt that this was the first time someone took their concerns seriously:

It was the first time someone had really listened to me as I voiced my concerns about my baby not putting on weight and took the time to watch me try to feed without hurrying me or intervening too much. (SP 7)

This non-judgemental support was important for women whose breastfeeding journey had not progressed as they expected, with one woman describing the way the counsellor helped her to “overcome guilt” for not breastfeeding:

They helped me in such a caring nature without prejudice or judgement, it helped me deal and overcome personal guilt about not being able to breastfeed. (SP 24)

Some women identified the qualities of a good peer counsellor, which included being a good listener, being kind and respectful.

I was met by a lovely breastfeeding counsellor and ushered into a private corner. She was kind, a good listener, not pushy and very respectful e.g. asking if I would mind feeding in front of her, shutting blinds and curtains, letting me decide what I wanted to do next, offering a drink. I warmed to her very quickly and really appreciated not being rushed – when you are that stressed and sleep deprived you need time to think, respond and make decisions. (SP 8)

Others made mention of the training and experience of ABA counsellors and the difference this made to the consultation:

I spent hundreds on private lactation nurses and nothing compares to the ABA. You can clearly see that their training and expertise is of another level, but combined with experience, empathy and understanding it brings their services to another level. (SP 25)

### The importance of face-to-face, practical and emotional support

Many women commented on the opportunity to sit face-to-face with a peer support counsellor throughout a full breastfeed and to receive practical support. The drop-in service provided face-to-face support alongside access to practical aids such as electronic breast pumps, which were available for hire onsite. This allowed women to leave the centre with information, suggestions and equipment. The suggestions provided by the peer supporters were described as “useful tricks and tips” provided for free.

Being able to sit in a private space with “another mother” who had additional experience and knowledge was one of the most valued things about the drop-in service. Many women mentioned the importance of face-to-face support rather than telephone support:

My son’s tongue and lip tie was finally picked up. It is extremely important these services are face-to-face as there are some things that can’t be done or seen over the phone. (SP19)

The opportunity to receive face-to-face support was viewed as one of the most beneficial aspects of the drop-in service:

The most helpful part of my experience at the breastfeeding lounge was that someone could actually see what my baby was doing at the breast and see her attachment. This would not have been achieved over the phone or by email. (SP 29)

Watching an entire feed seemed to be very important to women, particularly in a relaxed environment. The social nature of a face-to-face interaction seemed to be compatible with effective support for breastfeeding as a ‘social’ activity, allowing women to sit, talk and have a “cuppa”.

The calmness of the environment and the ability to wait until the baby was ready to feed was also a bonus. Women commented that the drop-in service felt like a safe place to go where they were exposed to other women who were experiencing difficulties. Women said they found this helpful, with one woman explaining:

Seeing other mothers going through difficulties made it much easier for me to persevere with breastfeeding. (SP39)

Interacting with other women who were on a similar journey highlighted the benefits of group interactions for sustained breastfeeding. Referrals to local ABA groups were identified as enabling ongoing breastfeeding following their visit to the centre:

We joined the ABA then and there and felt it was an absolute bargain for the service we received. I then went on to join and really enjoy my local ABA mothers group... I went away feeling more confident and with a whole lot of strategies to try. (SP7)

Beyond practical and informational support, the peer support counsellors at the drop-in service also offered emotional support, which women described as “boosting” or “lifting” their confidence. In addition to boosting their confidence, emotional support was identified by women as a positive outcome of their drop-in visit:

The support provided was very helpful but they also helped with my emotions as I was a first time mum. I only stopped breastfeeding my son 3 weeks ago and he turned 18 months old today. The first time I attended, which was not a drop-in day, I was extremely emotional and struggling with getting my son to latch. The lady helped me and if it hadn’t been for her I may have stopped breastfeeding my son. (SP 41)

Some women discussed feeling that their mental health was deteriorating, and the service helped them when they were “… becoming anxious and depressed …” (SP 25). The emotional support increased women’s feelings of confidence with their chosen feeding option. According to survey respondents, the peer counsellors reportedly focused their discussion on the woman’s needs and her unique situation rather than being focussed on their own agenda. One woman offered the following account:

They didn’t advertise the “breast is best” slogan in my face while they tried helping me with my baby during the consult, which was a breath of fresh air. At the time my baby was crying at the top of her lungs because she was hungry and wanted milk but just wouldn’t latch on so I gave her a bottle instead. I thought they would frown upon it and judge me and use the slogan about “the breast being the best”. They were so understanding and told me that giving her a bottle would be fine and it wasn’t at all my fault or baby’s, I was reassured that I was trying and doing my best. (SP25)

### The need for ongoing, free access: “I recommend it to all mothers”

Many of the respondents stated that following their experience, they recommended the ABA and the drop-in service to “everyone”. Women promoted the service to their friends and family:

It is a fabulous service that I recommend to all mothers that I meet who are experiencing any difficulties in feeding their babies. (SP5)

At the time of the survey, the drop-in service had stopped operating due to ongoing funding uncertainty. Some of the respondents were aware that the drop-in service had closed and expressed their disappointment that the service was no longer available to breastfeeding women. Many respondents described the service as “essential”, and some were worried about access to support with their next baby:

I am fairly terrified that I am pregnant again and won’t have access to this support the second time around! (SP2)

The fact that the service was free for members of ABA was appreciated. Women who were not members of ABA were also able to access the service but were encouraged to join ABA to ensure ongoing support. Some women stated that they liked the service so much they would have paid if needed:

I attended as my baby wouldn’t latch on properly and was not putting on weight. I was worried I had low milk supply … It was helpful that the service was free although I would happily have paid. (S P8)

Other respondents felt that they were misled that the service was free when in reality they were expected to join the ABA for ongoing access. Participants stated that this misinformation mostly came from health professionals suggesting it was a free service when they may not have been aware that it was only a free service for members.

I didn’t like being told that it was free but then had to sign up for the ABA membership which cost money to use the lounge. (SP20)

There was the suggestion from one respondent that this type of service should be readily available for everyone and should be provided by the health department, as the following quote describes:

The early childhood centre did not have a breastfeeding clinic (unlike the centre I visited with my first child) and I was told to see the clinic at the ABA. On arrival the counsellors were really approachable and friendly and calm. They were busy seeing to two other mums but got to me as soon as possible. I was very surprised to find out that I had to join as a member of the ABA to get any support. I felt that this was something that the early childhood centre should be providing free of charge. But I was glad that I was able to get some help and guidance regardless and understood that the ABA was not funded by Medicare or the health department. (SP35)

In particular, respondents identified wider benefits to society from a volunteer service such as this and the implications from its closure.

I was becoming anxious and depressed. If I hadn’t been to this service, I would have been diagnosed with postnatal depression which poses another whole set of stresses upon my family. These services need to be open and kept open as it benefits society and the government as tax payers as it becomes cost effective - umbrella affect. (SP25)

## Discussion

This study has considered the experiences of women attending a drop-in service run by the ABA. Participants shared their journey prior to the drop-in service and reported on the benefits of having access to this service. Quantitative analysis revealed that participants attended the drop-in service with predominantly small infants. Prior to attending the participants reported that some health professionals had been particularly unhelpful when seeking support for breastfeeding difficulties. Restricted tongue movement was suspected in a high number of infants brought to the drop-in service. In the qualitative responses, women highlighted the aspects of the service which were most valuable to them. These included: access to free face-to-face and non-judgemental peer support; having someone to ‘sit through a breastfeed’ and feeling listened to in a safe space. Many women described the service as the last resort in their breastfeeding journey. Participant responses confirmed that this drop-in service filled a gap in current service provision. Concerns expressed about the service closure highlighted the perceived value of this service and the fact that there were no similar style services available in the State.

### Inadequate health professional support

Participants in this study revealed that they had sought help from many health professionals before finding out about the ABA drop-in service. A number of participants rated GPs, paediatricians and child health services as the least helpful sources of support. When identifying the most helpful sources of breastfeeding support, participants in this study identified trained peers, such as the local ABA counsellor, or ABA 24 h Helpline, or health professionals with additional breastfeeding education (such as lactation consultants) as best. Professionals with formal and targeted education in breastfeeding support, such as Baby Friendly Health Initiative training, have been shown to provide up-to-date advice and support to breastfeeding women [18]. Embedding dedicated breastfeeding education into the health care system [19] and including greater access to peer support counsellors [13], may improve the appropriateness of breastfeeding support for women.

In particular, participants identified the failure of health professionals to detect infant tongue-tie as a breastfeeding related problem, prior to visiting the drop-in service. Peer counsellors at the drop-in service listened to women and referred to appropriate health professionals as necessary. The women in a study by Edmunds and colleagues [20], described difficulties trying to obtain diagnosis and treatment of their infants with tongue-tie. Ganesan and colleagues [21] found that many health professionals have limited knowledge about tongue-tie. Medical practitioners have consistently been shown to disregard tongue-tie and deny the impact on breastfeeding problems [22, 23]. Yet women in this study reported decreased pain with breastfeeding, and improved satisfaction, after the release of their infant tongue-tie. Whilst the counsellors at the drop-in service were not able to diagnose tongue-tie, simply listening to women and providing face-to-face, non-judgemental support, appeared to enable referral to appropriate diagnostic and treatment services and the resolution of difficulties caused by tongue-tie.

### Face to face, non-judgemental, reactive peer support

In this study, one of the dominant themes in the extended responses from women was the benefit of face-to-face peer support. The provision of support from another individual who is considered an ‘equal’ represents the foundation of breastfeeding peer support [24]. This form of support has been well established as positively impacting on breastfeeding rates [9] and satisfaction with support [25]. However, there have been debates over whether breastfeeding peer support is as effective when provided over the telephone [26] or face to face [27].

As discussed in the introduction to this paper, the most recent Cochrane review of breastfeeding support confirms that face-to-face support has a larger impact on exclusive breastfeeding rates, when compared to other methods of support, such as telephone only. Support provided by peers has also led to greater impact on breastfeeding continuation [9]. In a systematic review of the impact of peer support on breastfeeding duration, Jolly and colleagues revealed that higher intensity breastfeeding peer support interventions, or more frequent visits, led to higher rates of breastfeeding [28]. Yet the evidence for breastfeeding peer support is mixed with the Jolly and colleagues, systematic review reporting that proactive interventions, including antenatal and postnatal contact, are less effective than interventions that focus solely on the postnatal period [28].

In our study, breastfeeding peer support was ‘reactive’ support, provided in response to need, rather than ‘proactive’ [29], and in most cases was provided at a high stress access point during the postnatal period. Trickey [29] conducted an analysis of four key reviews on breastfeeding peer support and concluded that ‘proactive’ support yields the greatest improvements in breastfeeding rates. Forster and colleagues [15] recently shared the results from a proactive telephone-based peer support intervention in Australia which showed that proactive support can positively impact breastfeeding duration rates. The recent Australian National Breastfeeding strategy [30] promoted the need for proactive skilled support to be offered to breastfeeding women.

Subgroup analysis of the latest Cochrane review (2017), confirmed that there is a lack of sufficient studies for comparison of proactive versus reactive breastfeeding support, because too few studies have reported on reactive support [9]. Examples of the outcomes from reactive peer support services, such as drop-in services, are rare. The service we studied offered breastfeeding support at, what participants described as, a critical time period, which for some meant the difference between ceasing and continuing. This experience resonates with earlier work where women have described the importance of having access to reactive services and ‘the right help at the right time’ [13].

McLachlan and colleagues [31] conducted a large randomised controlled trial of a reactive breastfeeding support drop-in service, in combination with a proactive Maternal Child Health home visiting service, to determine if these interventions would improve breastfeeding rates. The drop-in services were established to provide both peer and professional support for women. The study reported that two of the drop-in services could not recruit peer supporters. One service that was able to recruit a peer supporter was located in a shopping centre and was poorly attended by women. The public location of one drop-in service, i.e. a shopping centre, and/or the very visible nature of another service i.e. the front window of a café, were considered to be possible factors in the low attendance at drop-in [32].

Previous research has established maternal preference for face-to-face support from a trained peer when ‘troubleshooting’ any problems beyond the early establishment phase of breastfeeding [27, 33]. Women often seek to avoid the perceived scrutiny [8], surveillance [34] communication style [35] and time pressures [8, 36] consistent with fragmented health professional interactions.

### Feeling listened to in a safe space

The availability of untimed interactions in a ‘mother friendly’ environment where the woman can decide on the frequency of contacts is highly desired by women [37]. Women in this study appreciated the fact that consultations with the peer counsellor were not timed so they could sit through a full feed. The desire to have someone ‘sit through a feed’ resonates with previous work [8, 35, 38]. Time for listening has been found to be an important component of confidence building for breastfeeding women [39]. Yet the opportunity for a health professional to ‘sit through a feed’ is seldom available in the early establishment phase of breastfeeding, especially on busy postnatal hospital wards [38, 40]. Although there are increasing opportunities for women to have access to relationship-based care through continuity of midwifery care with a known midwife [13].

Quinn and colleagues, in their study of women’s experience of breastfeeding support groups, also identified the importance of a ‘safe’ space to access support [27]. Having a service which women could return to as often as they liked, which was ‘safe’, and which was confidence enhancing rather than demoralising, offering choices rather than prescriptive advice, was highly valued by women. In this context it is concerning that due to funding shortfalls, this drop-in service was forced to close. An ABA funded breastfeeding centre continues to run in another Australian State, Victoria, with funding from that State Government [41].

### The last resort - supporting service sustainability

The service we explored reportedly gave participants confidence partly because the service provider, the Australian Breastfeeding Association, was available for their whole breastfeeding journey. The options for women to join the ABA and attend classes; use the telephone support; attend group meetings; or access online options for support; meant that the service reflected the realities of breastfeeding. Instead of a ‘one size fits all’ approach the service offered multiple options to suit individual preferences. In previous research, women have indicated the importance of being able to access support at the time they need it most, which can be from a peer counsellor or a midwife that has developed a professional relationship [13]. The availability of a 24/7 peer support telephone helpline allows women to seek appropriate support when dealing with breastfeeding challenges [16]. However, this is only part of the picture as women in this study indicated that they also wanted access to face-to-face support for visual confirmation that their technique, or infant behaviour, was normal, as well as for emotional and social support.

McLachlan and colleagues (2016) questioned the sustainability of ongoing drop-in provision when attendance is a fluctuating factor, which can compromise the viability of a service. Drop-in provided by volunteer peer counsellors is one way of reducing costs for supporting breastfeeding women however, volunteer peer counsellors are a transient population, who may also need to work and generate an income [31]. Thompson and colleagues [42] report on a peer support model in the UK called the ‘Star Buddies’ programme which includes payment for trained breastfeeding peer counsellors. In our study, peer support drop-in, provided by ABA, was utilised by women, but was closed due to cost saving strategies in an organisation which relies on government support from year to year [43]. The decision to close this service reflects a concern about the sustainability of an onsite face-to-face service run by volunteers.

New modes of engaging with women are gaining in popularity, such as using an online service delivery model [44]; Facebook groups [45]; text message interventions [46]; Skype and zoom possibilities for face to face support; mobile phone applications; and the use of video diaries [34]. Many of these alternative modes of support are also available for the full breastfeeding journey and offer women multiple methods of gaining access to peer support. The future of breastfeeding support must include online face-to-face opportunities, using Skype or ‘face time’ technology, to enable a peer counsellor to ‘sit through an entire feed’ and provide real-time face-to-face breastfeeding support alongside the provision of a 24/7 telephone helpline.

This study highlights some of the important components of peer support which women value, such as having access to trained counsellors, who have the capacity to provide non-judgemental, face-to-face support, who can sit through a feed, in a space that is ‘safe’, and who can enhance a woman’s confidence with breastfeeding over the course of her full breastfeeding journey. We agree with Cramer et al. [32] that the key factor in providing breastfeeding support is for communities to utilise existing services, such as the ABA, and implement interventions which provide maximum benefit for breastfeeding women, infants, and their families.

### Strengths and limitations

One of the strengths of this work is the presentation of women’s experience of attending an ABA run drop-in peer support service which is an innovative, but rarely available, service model. This was a small cohort of women, in one locality in NSW, with most women who accessed the service living within a 30-min radius. The sample was self-selected, and it is therefore possible that the respondents were mostly those who had a positive experience of the service. Some of the respondents were also aware that the drop-in service had closed, and their responses may have been motivated by a desire to ensure the service re-opened. This potential volunteer bias has implications for the generalisability of this study.

## Conclusions

This study revealed the experiences of women who attended a peer support drop-in service provided by the Australian Breastfeeding Association. Women described the importance of the availability of a drop-in service which was dedicated to breastfeeding support. Women highlighted the aspects of the service, provided by ABA peer counsellors, which were most beneficial, such as non-judgemental communication, confidence building, sitting through a feed, and the focus on listening and providing practical support. Many participants commented that the service was pivotal to enabling their continued breastfeeding and many expressed disappointment that the service was now closed. Low cost, face-to-face, emotional and practical support for breastfeeding women may increase the detection of breastfeeding difficulties and lead to longer durations of breastfeeding. Further research is needed to explore the impact and feasibility of cost-effective online face-to-face options for reactive drop-in peer support counselling, and the impact this has on women’s experience, confidence with breastfeeding, and achievement of personal breastfeeding goals.

## Data Availability

The datasets used and/or analysed during the current study are available from the corresponding author on reasonable request.
